# Relevance of regulatory T cell promotion of donor-specific tolerance in solid organ transplantation

**DOI:** 10.3389/fimmu.2012.00184

**Published:** 2012-07-13

**Authors:** Pervinder Sagoo, Giovanna Lombardi, Robert I. Lechler

**Affiliations:** ^1^Department Transplantation, Immunoregulation and Mucosal Biology, MRC Centre for Transplantation, King's College LondonLondon, UK; ^2^NIHR Biomedical Research Centre, Guy's and St. Thomas' NHS Foundation Trust and King's College LondonLondon, UK

**Keywords:** donor-specific, regulatory T cells, operational tolerance, indirect pathway, antigen-specific, direct pathway, linked suppression, alloantigen

## Abstract

Current clinical strategies to control the alloimmune response after transplantation do not fully prevent induction of the immunological processes which lead to acute and chronic immune-mediated graft rejection, and as such the survival of a solid organ allograft is limited. Experimental research on naturally occurring CD4^+^CD25^high^FoxP3^+^ Regulatory T cells (Tregs) has indicated their potential to establish stable long-term graft acceptance, with the promise of providing a more effective therapy for transplant recipients. Current approaches for clinical use are based on the infusion of freshly isolated or *ex vivo* polyclonally expanded Tregs into graft recipients with an aim to redress the *in vivo* balance of T effector cells to Tregs. However mounting evidence suggests that regulation of donor-specific immunity may be central to achieving immunological tolerance. Therefore, the next stages in optimizing translation of Tregs to organ transplantation will be through the refinement and development of donor alloantigen-specific Treg therapy. The altering kinetics and intensity of alloantigen presentation pathways and alloimmune priming following transplantation may indeed influence the specificity of the Treg required and the timing or frequency at which it needs to be administered. Here we review and discuss the relevance of antigen-specific regulation of alloreactivity by Tregs in experimental and clinical studies of tolerance and explore the concept of delivering an optimal Treg for the induction and maintenance phases of achieving transplantation tolerance.

## Introduction

Transplantation presents a life-saving treatment for patients with end stage organ failure, however the success of this procedure is restricted by the recipient immune response directed against donor graft alloantigens and the clinical caveats associated with immunosuppressive drugs aimed at controlling this immune response. Current strategies for clinical management of transplant recipients using sustained immunosuppression do not fully prevent induction of the immunological processes which lead to graft rejection, namely chronic allograft failure, and as such the survival of a solid organ allograft is limited (Meier-Kriesche et al., 2004b; Lamb et al., 2010). Whilst early attrition rates for solid organ transplantation have significantly improved over the last few decades, attributed to reduced ischemia times, improved clinical procedures and patient care management, long term survival of allografts have remained relatively unchanged, requiring the majority of patients to have further organ transplants (Meier-Kriesche et al., 2004a; Lodhi et al., 2011). As this inevitably results in an escalating shortage of donor organs, there is a pressing need to develop an alternative method to control the alloimmune response which can establish stable long-term graft acceptance through induction of donor-specific immunological tolerance.

Transplantation tolerance can be defined as a state of immune unresponsiveness, downregulation or deviation of an immune response to an inflammatory situation or insult such as that generated by the recipient immune response following transplantation. Decades of experimental research have identified that mechanistic bases of immune tolerance may be through processes of deletion, anergy, antigen sequestration or immunological ignorance, and also the focus of this review, through processes of active regulation. Implementing mechanisms of immune regulation for tolerance induction are more desirable as an approach as it will, in principle, provide a mechanism which can adapt to the dynamic and evolving immune response post-transplantation. Amongst the T cell subsets with immunomodulatory properties, the regulatory roles of thymus derived CD4^+^CD25^high^FoxP3^+^ naturally occurring regulatory T cells (Tregs) have been recognized for many years and substantial research efforts have sought to exploit their suppressive functions to deliver a tolerogenic cell therapy for transplantation (Hippen et al., 2011; Lombardi et al., 2011). This transition to the clinic has been facilitated by the significant progress made over the last 5 years in identification of further markers to delineate stable suppressive Treg subsets, such as CD45RA, CD161, CCR6, and low expression of IL-7 receptor α chain CD127, in addition to previously described expression of transcription factor Forkhead box p3 (FoxP3), CTLA-4, GITR, and CD62L (Liu et al., 2006; Miyara et al., 2009). Recently, their development as a cell therapy has been translated to clinical hematopoietic stem cell transplantation settings (Sakaguchi, 2004; Sagoo et al., 2008) and use in phase I and II clinical trials are showing tentative yet encouraging results in terms of both safety and efficacy (Brunstein et al., 2011; Di Ianni et al., 2011). The main therapeutic approach currently in use is to infuse freshly isolated or *ex vivo* polyclonally expanded Tregs into graft recipients with an aim to provide a more favorable *in vivo* balance of T effector cells to regulatory cells. However, our current understanding of the alloimmune response suggests that regulation of donor-reactive immunity primed by specific pathways of alloantigen-presentation following transplantation may be central to achieving long-term or indefinite graft survival (Nepom et al., 2011; Wood et al., 2011). This concept is now being supported by mounting experimental evidence from basic and clinical studies, which indicate that the next stage in optimizing translation of Tregs to solid organ transplantation will be through the refinement and delivery of donor alloantigen-specific Treg therapy.

This review article discusses the relevance of antigen-specific regulation of alloreactivity by Tregs and explores the concept and goal of defining an optimal Treg for the prevention of transplant rejection and induction of organ transplant tolerance. We identify the main features of the immune response which Tregs need to control by firstly reviewing evidence for the induction and temporal pattern of the alloimmune response, in terms of alloantigen presentation and allopriming following transplantation, and the resulting effector mechanisms of graft rejection. We then review evidence for the association of Tregs and Treg-mediated donor-specific immune regulation in clinical transplantation with particular focus on data emerging from the study of operationally tolerant transplant recipients. After reviewing these findings we then discuss the mechanistic bases of tolerance induction by antigen-specific Tregs, and the requirements of an optimized Treg to improve the success of this approach for the induction and maintenance phases of achieving donor-specific tolerance.

## The alloimmune response

**Figure 1:**
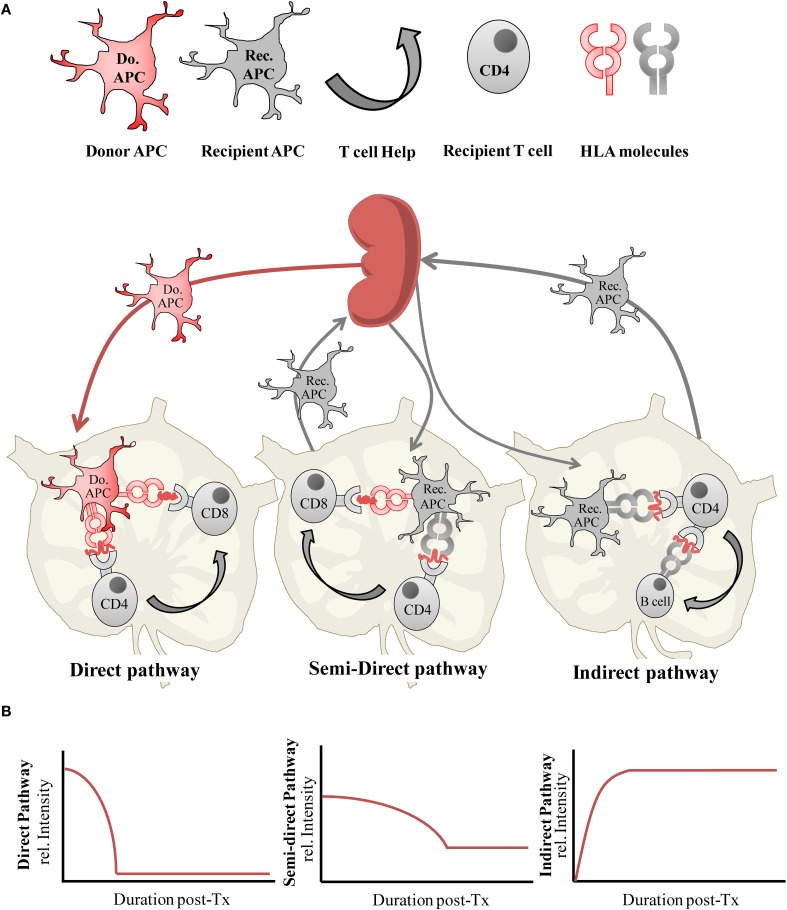
**(A)** Alloantigen presentation via the direct, semi-direct and indirect pathways following organ transplantation, and **(B)** the relative intensity of each antigen-presentation pathway during the post-transplantation (post-Tx) period.

Induction of the adaptive immune response to an allograft begins with recognition of alloantigen by recipient T cells which is now well characterized and known to occur through three main processes known as the direct, the indirect, and the semi-direct pathways of antigen presentation. The relative contributions of the direct and indirect pathways of alloantigen presentation toward graft rejection have been reviewed in detail elsewhere (Afzali et al., 2007; Gokmen et al., 2008), however the key questions we examine here are whether the differential activity of these alloantigen presentation pathways are associated with transplantation tolerance, and whether their activity is modulated though a process of active regulation which may otherwise be achievable using alloantigen-specific Treg therapy. Our understanding of factors such as the temporal activity and intensity of alloantigen presentation pathway activity, and resulting alloimmune priming following transplantation is integral to identifying the specificity of the Treg required and the time or frequency at which it needs to be administered to deliver an optimized and targeted therapeutic. We therefore begin by providing a brief updated overview of allorecognition, which is summarized in Figure 1A.

### Pathways of alloantigen presentation

The *direct pathway* of alloantigen presentation is so named as intact allogeneic major histocompatibility complex (MHC) molecules expressed by donor allograft derived cells are directly presented to recipient T cells. The most potent driver of CD4^+^ and CD8^+^ T cell responses with specificity for alloantigen presented by the direct pathway is through the migration of donor antigen-presenting cells (APCs) from the allograft to the secondary lymphoid organs (Larsen et al., 1990). Here, donor MHC alloantigens are recognized by alloreactive T cells which are estimated to be of a relatively high endogenous frequency of between 1:100 and 1:100,000 T cells in humans (Hornick et al., 1998; Game et al., 2003; Benitez and Najafian, 2008), and even higher (1:10) in mouse (Suchin et al., 2001). As such, they are able to elicit a vigorous inflammatory T cell response toward the allograft resulting in early or acute rejection. This pre-existing population of T cells with specificity for the direct pathway is a long-standing conundrum in immunology, as recipient T cell recognition of foreign MHC molecules which have not previously been encountered in the thymus violates the rules of self-MHC restriction therefore, direct allorecognition may be attributed to cross-reactivity, namely the ability of self-MHC restricted T cell T cell receptors (TCRs) to recognize polymorphic residues on foreign MHC through structural similarities between donor and recipient MHC molecules (Lombardi et al., 1991; Lechler et al., 1992), although evidence exists which favors the hypothesis that the peptide primarily determines the diversity of the T cell response (Weber et al., 1995). The latter may also explain the occurrence of alloreactivity when donor and recipient MHC are structurally dissimilar. Based on the nature of direct pathway allorecognition (Gras et al., 2011), the direct pathway alloreactive T cell compartment is predicted to arise equally from within either naive CD45RA^+^ or memory CD45RO^+^ T cell compartments and is polyclonal in nature (Merkenschlager and Beverley, 1989; Lombardi et al., 1990). Macedo et al. have recently provided further evidence for this by studying effector functions (IFNγ production), proliferation and precursor frequencies in isolated human CD4^+^ and CD8^+^ naive and central/effector memory T cell subsets, in response to stimulation with allogeneic peripheral blood mononuclear cells (PBMCs; Macedo et al., 2009). These characteristics have particular relevance to the development and application of effective immunosuppressive approaches to control the direct alloresponse. A lesser described stimulator of the direct alloresponse is that of the presentation of allogeneic MHC by non-professional antigen presenting cells such as activated donor endothelium or epithelium within donor tissue. While several studies have shown this may be able to stimulate T cells requiring lower costimulatory signal thresholds, such as memory T cells (London et al., 2000; Berard and Tough, 2002), other work has shown this may not necessarily result in productive stimulation of alloreactivity (Marelli-Berg et al., 1996).

The *indirect pathway* of alloantigen presentation occurs when recipient bone-marrow derived APCs capture, process, and present allogeneic MHC determinates to recipient T cells. In this pathway, alloantigen may be acquired from the circulation from shed donor graft material, collected by recipient APCs trafficking through the allograft, or through the phagocytosis of donor APCs that have migrated to the draining lymph nodes. As stimulation of this pathway is dependent upon the limitless supply of graft-derived antigens, it is initiated immediately post-transplantation and sustained throughout the life of the graft. The pre-existing endogenous frequency of alloreactive T cells with specificity for the indirect pathway is detected to be much lower than that of the direct pathway, with a range in frequency of 1:100,000–1:1,000,000 T cells (Hornick et al., 2000; Baker et al., 2001a). Although this antigen presentation pathway becomes active immediately following transplantation, the initially low frequency indirect alloresponse is not generally considered to be of sufficient intensity to be the main stimulus of early or acute graft rejection in a clinical setting, although in some experimental models of graft rejection described later it has been shown to induce acute rejection. Instead, the continuous and progressive priming of the immune response to indirectly presented alloantigens is thought to gradually amplify effector T cell responses with indirect allospecificity to culminate in chronic immune-mediated rejection (Vella et al., 1997; Hornick et al., 2000; Baker et al., 2001a; Gokmen et al., 2008).

The *semi-direct pathway* is the most recently described pathway of antigen presentation and occurs when intact allogeneic MHC:peptide complexes are captured from donor cell membranes by recipient APCs and incorporated, with maintenance of sufficient molecular and structural integrity to prime recipient T cell alloresponses to the direct pathway (Herrera et al., 2004; Smyth et al., 2006; Riond et al., 2007; Smyth et al., 2007). There is, therefore, the potential for any given APC to simultaneously present alloantigen both via the direct and indirect pathways. In addition to dendritic cells (DCs) and macrophages, a significant proportion of B lymphocytes have also been shown to acquire allogeneic MHC molecules, and this process is also now known to be bidirectional whereby cells of donor origin can also capture and present recipient derived MHC complexes (Brown et al., 2008, 2011).

The ability of the semi-direct pathway to link both the direct and indirect pathways of alloimmune priming of T cell responses may simplify the matter of selecting an appropriate allospecific Treg to regulate the alloreponse by permitting linked or bystander suppression by allospecific Treg, a concept which we return to later. The semi-direct pathway, therefore, illustrates that neither pathway of alloantigen presentation is mutually exclusive post-transplantation. This is further revealed by studying the mechanisms, kinetics, and altering intensity of the alloimmune response.

### Dynamics of alloantigen presentation

An important aspect of administering optimized donor-specific Treg cell therapy is to determine when to deliver their immmunoregulatory effects *in vivo*. A sensible presumption would be to apply them in advance of or simultaneously to the induction of alloantigen presentation pathway activities, to counteract the allopriming effect. Our understanding of the dynamics of the alloimmune response is provided in part by studying the survival and trafficking of donor and recipient APCs *in vivo* but is also revealed more directly by experimental and clinical studies reporting on the duration and intensity of both direct and indirect pathway primed T cell alloresponses following transplantation (Figure 1B).

The contribution of alloimmune priming by the direct pathway was first demonstrated by seminal experiments examining the effects of donor allograft passenger APCs on kidney allograft survival (Lechler and Batchelor, 1982a). Several studies have since confirmed that abrogation of the direct pathway typically results in a prolongation of graft survival rather than achieving an outcome of true tolerance (Garrod et al., 2010; Fernandes et al., 2011; Gupta et al., 2011), suggesting it is not the only driver of graft rejection. Due to the clearance of donor passenger APCs, the direct alloresponse it is thought to be a relatively short-lived (Lechler and Batchelor, 1982a,b; Hornick et al., 1998), allowing indirect allospecific T effectors responses to dominate. Clearance of donor APCs has recently been shown to be a highly efficient process mediated by recipient cytotoxic lymphocytes (CTLs; Laffont et al., 2006) and natural killer (NK) cell killing, the latter of which can efficiently remove allogeneic donor APC introduced by adoptive transfer or by a skin allograft within hours post-transplantation, to limit any consequent priming of direct pathway alloreactive effector T cell responses (Laffont et al., 2008; Garrod et al., 2010). However, the discovery of the semi-direct pathway implies that the direct pathway is not completely inhibited by this process. Therefore, these data clearly suggest that targeted approaches to control both the direct pathway, perpetuated by the semi-direct pathway, and the indirect pathway may be better able to deliver tolerance induction.

Recent data supports this view by providing evidence of the presentation of donor alloantigen by the semi-direct pathway for prolonged periods post-transplantation. In a rat model of complete MHC mismatched (LEW→BN) liver transplantation, Toyokawa et al. were able to detect donor MHC Class II L21-6^+^ CD11c^+^ cells within allografted tissues up to 200 days post-transplantation (Toyokawa et al., 2008), but surprisingly found they disappeared much earlier when grafts were performed into recipient animals pre-depleted of macrophage and DC compartments using clodronate liposomes. While the authors speculated that this early loss of cells expressing donor MHC class II may be due to lack of a survival advantage conferred by recipient APCs through microenvironment conditioning, other studies of surface MHC transfer suggest that the prolonged persistence of donor MHC expression of CD11c^+^ cells is more likely to be due to the semi-direct pathway. In a mouse kidney graft model of spontaneous tolerance (DBA-2→C57BL/6), Brown et al. were able to demonstrate activity of the semi-direct pathway as early as 8 days post-transplantation by counter-staining lymphoid tissues for both recipient and donor MHC class II expression (Brown et al., 2008). They were able to detect a surprisingly high proportion of APCs with I-A^d^ (DBA-2 MHC class II) and I-A^b^ (C57BL/6 MHC Class II) expression (~30%), which remained detectable for extended periods of over 80 days post-transplantation. In the study by Tokoyawa et al., the semi-direct pathway is, therefore, a more likely explanation for prolonged direct pathway donor alloantigen presentation, particularly as in this same study they also detected upregulation of MHC class II expression by donor allograft epithelial and endothelial cells during inflammation, which could have provided a continuous source of donor alloantigen. This would however need to be confirmed by staining for both donor and recipient MHC expression.

Although CD4^+^ T cells with direct allospecificity are well placed to provide help to alloreactive CD8^+^ T cells, through the likely 3-cell clusters formed between direct alloreactive CD4^+^ and CD8^+^ T cells interacting with a donor-derived APC expressing both allogeneic MHC class I and II, there must be an alternative provision of help for CD8^+^ T cells, as otherwise the clearance of donor passenger APCs would result in a parallel reduction of alloreactive CD8^+^ CTL responses. This long-standing conundrum is partly resolved by demonstration of a helper T cell-independent mechanism of CD8^+^ T cell alloactivation (Jones et al., 2006) and partly by the provision of T cell help via CD4^+^ T cells with indirect allospecificity (Lee et al., 1994). Precisely how an indirect alloreactive CD4^+^ T cell with self-MHC restriction would encounter a direct allospecific CD8^+^ T cell interacting with a cell expressing intact allogeneic Class I MHC, is resolved by the semi-direct pathway where an APC can present both allogeneic class I and processed allopeptides in the context of Class II self-MHC. Fischer et al. have shown that combining tolerogenic conditioning of recipient murine DCs through Rapamycin drug treatment, with DC capture of intact MHC Class I from allogeneic cell lysates, results in alloantigen presentation via the semi-direct pathway, and drives regulation of direct alloreactive CD8^+^ T cell responses *in vitro* and *in vivo*. This study indicates the potential of this pathway in mediating effective immune regulation as well as alloimmune priming (Fischer et al., 2011). Whereas presentation of donor-derived MHC Class I via the semi-direct pathway may remain sustained, as stable graft function develops through sustained immunosuppression or developing immune regulatory processes, presentation of MHC Class II via this pathway may play a lesser role during the post-transplant period as it's sources and activation-induced expression subside with diminution of the inflammatory microenvironment.

Combined, these data shed an alternative view on the role of donor APCs in priming the alloresponse early post transplantation, where their main effect may also be through the provision of an early and high density source of donor antigen to prime the indirect and semi-direct pathways. Activity of these two pathways has recently been demonstrated on a more direct visual basis by the detection of Yae (antibody with specificity for Class I H2-K^d^ peptide presented by I-A^b^ complex) and MHC class II I-A^d^ double positive APCs within lymphoid tissue of C57BL/6 (H2-K^b^) mice that have received a BALB/c (H2-K^d^) heart graft, demonstrating the capacity of recipient APCs to simultaneously prime both direct and indirect T cell alloresponses (Brown et al., 2011). The predominance of alloantigen presentation via the indirect pathway early in the post-transplantation period in addition to its more usual role in chronic rejection is now becoming a better established phenomenon.

In this respect, the contribution of the indirect pathway toward allograft rejection has been firmly established by studies using donor grafts from MHC class II^−/−^ mice, where rejection can be efficiently induced in the complete absence of direct pathway presentation of alloantigen (Auchincloss et al., 1993; Honjo et al., 2004). Graft rejection dependent on indirect pathway presentation of alloantigens or minor antigens is also now well described (Jurcevic et al., 2001; Sims et al., 2001; Smith et al., 2001; Fernandes et al., 2011), and can occur with little or no change (Garrod et al., 2010; Gupta et al., 2011) in the kinetics of graft rejection compared to when the direct pathway is also active. In support of these findings Brennan et al. have demonstrated the efficiency of the indirect pathway compared to the direct pathway of alloantigen presentation (Brennan et al., 2009). This study co-transferred murine CD4 and CD8 T cells with TCR transgenes conferring specificity for either the direct pathway (I-A^d^ and H2-K^d^, respectively), or the indirect pathway (H2-K^d^:I-A^b^ complex) into C57BL/6 mice (I-A^b^) which were then challenged with BALB/c (I-A^d^) heart or skin grafts. On adoptive transfer, T cells with indirect allospecificity proliferated with much more rapid kinetics compared to T cells with direct specificity, which was also reflected by endogenous alloreactive T cell populations. Using a murine transplant model Gupta et al. were able to measure the kinetics of alloantigen presentation though the direct or indirect pathways by grafting skin from a BALB/c or CB6F1 (C57BL/6 × BALB/c F1) mouse onto a C57BL/6.TEa.Rag2^−/−^ recipient mouse, in which the T cells have indirect specificity for I-E alpha peptide presented by C57BL/6 MHC class II I-A^b^ (Gupta et al., 2011). They found that on indirect presentation alone, where BALB/c donor alloantigens must first be processed via the indirect pathway for presentation to TEa T cells, rejection was delayed by 6 days compared to direct antigen presentation (endogenous expression of I-Eα:I-A^b^ complex by CB6F1 DCs), suggesting this short delay was caused by the time lag required for antigen processing and presentation by the recipient DCs before subsequent T cell proliferation. Although these findings concur with the concept that late graft rejection is associated with a gradually priming and maintenance of the indirect pathway, they fundamentally differ from the findings by Brennan et al., however these differences may be attributed to the allograft model or TCR transgenic T cells used, suggesting although these models are ideal for dissecting mechanistic basis of alloimmune priming, examining clinical data may be more informative for developing practical therapeutic strategies for intervention.

### Implications for donor-specific Treg therapy

The experimental findings described above underpin two main features of alloimmunity, firstly that the indirect pathway is active immediately following transplantation and secondly that the direct pathway can contribute toward the maintenance of the indirect pathway and may be active later in the post-transplantation period through the semi-direct pathway. It may, therefore, be tempting to speculate that Treg therapy would be best applied either pre-transplantation or at the time of transplantation to prevent any initial priming of memory alloreactive T cell responses, perhaps through modulation of APC activities (Misra et al., 2004; Mahnke et al., 2007; Herman et al., 2012). However, several recent studies have demonstrated that maintenance of alloantigen presentation throughout the lifetime of the organ transplant is also key to achieving tolerance, particularly so when mechanistically Tregs are involved.

Chiffoleau et al. have shown that a rat model of donor-specific heart transplant tolerance (fully MHC mismatched LEW.1W→LEW.1A) is associated with an expansion of splenic Tregs. In parallel, the study detected the persistence and even proliferation (possibly a consequence of the deoxyspergualin analogue tolerising protocol used) of donor DCs for over 100 days post-transplantation, which were restricted to the allografted tissue, achieving a localized tissue chimerism (Chiffoleau et al., 2002). Interestingly, this group found that pre-depletion of donor APCs from the heart allograft prior to transplantation by cyclophosphamide treatment, resulted in a reduction in splenic Tregs and abrogation of the tolerogenic effect. *In vitro* analysis further confirmed that CD4^+^ T cells from tolerised animals showed direct pathway donor-specific suppressive activity. Therefore, using this particular tolerising protocol, alloantigen presentation via the direct pathway was critical for induction of transplant tolerance and generation of donor-specific Tregs, suggesting this may be mechanistically a critical contributor toward the successful approach of using mixed-chimerism to induce tolerance to a solid organ transplant (Ko et al., 1999b; Andreola et al., 2011).

Several other studies have also shown that persistent presentation of donor alloantigen is also essential for the maintenance of solid organ transplant tolerance in rodent models. Hamano et al. showed that stable tolerance of a murine allogeneic heart graft induced by anti-CD4 monoclonal antibodies and donor-specific transfusion (DST) was lost when second donor-matched hearts were transplanted 200 days after the removal of the primary hearts, that is, once all donor-alloantigen had been cleared from the recipient mouse (Hamano et al., 1996). This implies that unlike the study by Chiffoleau et al., donor-microchimerism through the survival of donor APCs is not essential for maintenance of tolerance (Ko et al., 1999a), but rather that activation of semi-direct or indirect pathways of alloantigen presentation, by any source of alloantigen, may be more critical. This theory corresponds with evidence of the requirement of continuous presence of donor alloantigens for Treg survival in allograft tolerance models (Scully et al., 1994; Chen et al., 1996; Hamano et al., 1996). Two recent studies add further support to this concept by using miniature swine models of allotransplantation to study the stability of tolerance and the contribution of alloantigen presentation via the indirect pathway toward maintenance of tolerance (Okumi et al., 2008; Weiss et al., 2009). Okumi et al. were able to demonstrate that once a primary MHC class I mismatched tolerised kidney allograft had been removed and all endogenous donor alloantigen had been cleared, animals could be sensitized to reject a second identical donor graft by injection of donor MHC Class I peptides. However, immunization of long-term tolerised animals with donor MHC Class I peptides did not lead to the rejection of a primary graft, indicating that alloantigen presentation via the indirect pathway could both break tolerance and be crucial in the induction and maintenance of tolerance.

The relevance of these studies toward developing Treg therapy is challenging to conclude, largely because of the intricacies and variation within each experimental model, with respect to transgenic mice used or tolerising protocols applied, which may or not be associated with Treg mediated regulatory processes. What can be deduced is that for induction of long-term stable graft tolerance, active presentation of alloantigen is required, which over the life-time of an allograft will be mediated primarily by the indirect pathway. Sustained alloantigen presentation may function by promoting antigen-driven activation, expansion, or survival of *in vivo* induced or adoptively transferred donor-specific Tregs (Walker et al., 2003; Sanchez-Fueyo et al., 2006), which may in turn stimulate other alloantigen-specific immune regulatory processes, a concept which is explored in more detail in the following section.

## Donor-specific Treg control of alloimmune responses following transplantation

As well as their critical role in immune homeostasis and regulation of autoimmunity (Sakaguchi and Sakaguchi, 2005), Tregs can function to regulate the alloimmune response through a number of mechanisms which include release of immunosuppressive cytokines, modulation of APC and endothelial functions, or direct suppression of CD8^+^ and CD4^+^ T effector cells to summarize but a few (Shevach, 2009). Here we examine the requirements of Tregs to control several important aspects of the alloimmune effector response, outlining where the advantages of donor-specific Treg use for immune regulation may lie.

### Donor-specific Tregs deliver localised immune regulation

Targeting the immunoregulatory properties of Tregs to the sites of anti-donor effector responses would circumvent the issue of the relative paucity of Tregs, which would otherwise limit their efficacy *in vivo*. The effector arm of the indirect alloresponse is broad and can occur at remote sites to the donor allograft as discussed later, however as indirect allospecific T cells are unable to recognize the tissue allograft directly to mediate direct lysis or cell mediated immunity, that is in a completely MHC mismatched donor recipient setting, they would only potentially be able to directly damage the allograft by bystander killing. In contrast, direct allospecific T cells accumulate and act at the graft site through recognition of expressed intact MHC. An immediate advantage of transferring Tregs with direct pathway alloantigen-specificity would, therefore, be that they would naturally localize to the equivalent site of allospecific effector T cell priming to mediate their regulatory effects. Indeed, Tregs have been described to specifically accumulate at sites of alloantigen sources, alloimmune effector priming or target activity, to establish a state of local immune privilege (Golshayan et al., 2007, 2009; Dijke et al., 2008). In experimental models of allograft tolerance, Tregs with the ability to transfer tolerance to naive recipients have also been detected within draining lymph nodes and also donor allografted tissue (Graca et al., 2002). In a recent study by Heslan et al., analysis of T cells isolated from tolerised allografts, also capable of transferring donor-specific tolerance to naive recipients, showed skewed TCR Vβ repertoires which may reflect an accumulation of oligioclonal donor alloantigen specific regulatory T cells (Heslan et al., 2005). What many studies have been unable to demonstrate is whether Tregs are generated elsewhere and then migrate to the graft site from the periphery or whether they are induced within an allografted tissue itself. However these studies do highlight the major advantage offered by a therapeutic strategy to adoptively transfer donor-specific Tregs into transplant recipients, by allowing their immunomodulatory functions to be readily concentrated at the source of their cognate alloantigen expression and subsequent immune activation (Golshayan et al., 2007). We have recently demonstrated that transfer of human Tregs selected for direct pathway donor allospecificity are more effective at preventing rejection of a human skin graft in a humanized mouse xenograft model, compared to polyclonal Tregs (Sagoo et al., 2011). On studying early trafficking of adoptively transferred human Tregs (3 days), similar numbers of both allospecific and polyclonal Tregs were recruited to skin allografts, although a higher proportion of allospecific Tregs were found to be in contact with skin resident alloantigen (HLA-DR^+^) bearing donor cells. These data allow speculation that donor-specific Treg mediated suppression occurs primarily at sites of alloantigen expression and effector target tissue, and possibly acts through early interaction and modulation of APC function and effector cell recruitment, as implicated by other *in vitro* and *in vivo* studies (Golshayan et al., 2009; Herman et al., 2012). Analysis at later time points (4 weeks) showed significantly higher numbers of allospecific Tregs were present in allografted tissues, which concurs with the hypothesis that antigen-driven expansion or survival of allospecific Tregs had occurred (Walker et al., 2003; Sanchez-Fueyo et al., 2006), and which may contribute to the improved efficacy of donor-specific Tregs in this model. These findings also resonate with functional differences detected between antigen-specific and polyclonal Tregs in other murine models of tolerance induction (Golshayan et al., 2007; Joffre et al., 2008; Tsang et al., 2009). In contrast, considerable success using polyclonal Tregs in preventing experimental graft versus host disease (GvHD) has also been demonstrated (Edinger et al., 2003; Trenado et al., 2003). The disparity between the relative efficacy of polyclonal Tregs in these transplantation settings may again be related to the localization of alloreactive responses, which during GvHD is more systemic and, therefore, equally amenable for polyclonal Treg mediated regulation.

The frequency and distribution of Tregs have also been studied in the context of clinical transplantation both in observational/association studies, and also in several studies extending to cellular function analysis of Treg mediated suppression, the latter of which is discussed later. As summarized in Table 1 (Columns A and C), several studies examining clinically stable allograft recipients, those undergoing rejection, and healthy “control” individuals, in general find no consistent differences in the frequencies of peripheral blood circulating levels of Tregs. Shan et al. (2011) have recently compiled a more comprehensive review of over 20 observational clinical studies which have examined the association of detected human Tregs with liver, heart, lung, and kidney allograft outcome. Their meta-analysis shows that elevated intra-graft Tregs detected by relative increase in FoxP3^+^ cells or quantitative mRNA expression expression could, in general, be positively correlated with improved graft function or outcome, whereas numbers of circulating Tregs, the most common method of analysis, could not be consistently correlated with outcome. This finding again reinforces the concept of targeting Tregs to the correct *in vivo* site for optimal alloimmune suppression. This is further supported by a study by Bestard et al. which found that renal transplant patients whom developed T cell hyporesponsiveness toward their donor after transplantation also had significantly higher levels of CD4^+^FoxP3^+^ cells within their allograft infiltrates compared to patients whom showed donor-reactivity (Bestard et al., 2007). Similar observations of elevated *foxp3* mRNA expression in allograft biopsies have also been made in combined bone marrow transplant (BMT) and kidney allograft patients whom develop operational tolerance (Kawai et al., 2008).

**Table 1:** **Evidence of donor-specific regulation by Tregs in clinical transplantation**.

		**Column A**	**Column B**	**Column C**
**Organ transplant; Reference**	**Study groups (*n* =) time post-tx^a^**	**Antigen-specific response studied & analysis method**	**Alloreactivity & evidence for Treg-mediated suppression**	**Summary**
**Kidney**; Akl et al., 2008	ST (15) 7.6 ± 3 years CR (15) 6 ± 3.7 years HC	***Direct pathway*** Responder: recipient whole PBMC Stimulator: Do, 3rdP PBMC Readout: proliferation, CFSE Assay for regulation: CD4^+^CD25^+^ Treg depletion from MLR Other assays: PBMC analysis flow cytometry	ST: All patients were donor (and 3rdP) hyporesponsive 11 of 15 patients showed Treg mediated donor-specific suppression 6 of 11 patients showed evidence of CD8 T cell donor-specific suppression by Tregs CR: Donor-hyperesponsive 4 of 15 patients showed non-specific Treg mediated regulation	Function Evidence of **Treg mediated donor-specific suppression** of CD4 and CD8 T cells in ~73% of stable patients Phenotype **Higher absolute number of PB CD4^+^CD25^hi^** ST vs. CR = *p* < 0.01 ST vs. HC = ns
**Kidney**; Velthuis et al., 2006	ST (33) > 5 years	***Direct pathway*** Responder: recipient PBMC Stimulator: Do, 3rdP PBMC Readout: ^3^H proliferation, Day 7 Assay for regulation: Isolated CD4^+^CD25^bright^ Treg titrated back into MLR cultures Other assays: PBMC analysis of CD4^+^CD25^bright^ Treg and *foxp3* mRNA	ST: 25 of 33 patients were donor-hyporesponsive 7 of 25 donor-hyporesponsive patients showed Treg mediated donor-specific suppression	Function Evidence of **Treg mediated donor-specific suppression** in ~20% of stable patients Phenotype **No difference in % CD4^+^CD25^bright^ or foxp3 expression** between non-hyporesponsive and hyporesponsive patients
**Kidney**; Velthuis et al., 2007	ST (6) 8 months-7 years	***Direct pathway*** Responder: recipient PBMC Stimulator: Do, 3rdP PBMC Readout: ^3^H proliferation, Day 7 Assay for regulation: Isolated CD4^+^CD25^bright^ Treg titrated back into MLR cultures	ST: 5 of 6 patients were donor-hyporesponsive 5 of 6 patients showed Treg mediated donor-specific suppression which was only detected when Tregs added to MLR at Day 5	Function Evidence of **Treg mediated donor-specific suppression** in ~83% of stable patients
**Kidney**; Baan et al., 2007	ST (11)^*^ 3–13 months HC (15) ^*^3/11 had previous AR episodes, only 5 patients used for functional studies	***Direct pathway*** Responder: recipient PBMC Stimulator: Do, 3rdP PBMC, TTOX Readout: ^3^H proliferation Day 7, IL-2 production in MLR Assay for regulation: Depletion of and add back of isolated CD4^+^CD25^bright^ Treg into MLR cultures Other assays: PBMC analysis of CD4^+^CD25^bright^ Treg	ST: 8 of 11 patients showed non-specific regulation of donor and 3rdP responses by Tregs by Treg depletion 2 of 5 patients showed complete suppression (non-specific) by Tregs by add of isolated Tregs 4 of 5 patients showed detectable TTOX response with Treg mediated regulation of responses	Function No evidence of direct pathway hyporesponsiveness or Treg mediated suppression of donor-specific responses Phenotype **No difference in % of CD4^+^CD25^bright^** in PB between ST and HC
**Kidney**; Hendrikx et al., 2009	Prospective study (79) Analysis pre-tx, 3, 6, and 12 months post-tx	***Direct pathway*** Responder: recipient CD4^+^CD25^neg/dim^ Stimulator: Do, 3rdP PBMC, TTOX Readout: ^3^H proliferation Day 7, IL-2 production in MLR Assay for regulation: Depletion and add-back of CD4^+^CD25^bright^ Tregs into MLR cultures Other assays: PBMC flow cytometry phenotype of Tregs	Evidence of significant Treg mediated donor-specific suppression compared to 3rdP only detected after 6 months post-tx	Function Development of **Treg mediated donor-specific suppression** within 1 year post-tx Phenotype **Decrease in % and absolute number CD4^+^CD25^bright^ Treg** post-tx
**Kidney**; Bestard et al., 2007	Prospective study (20); Patients treated with rATG induction, and MMF/SRL post-Tx. Analysis up to 34 months post-Tx	***Direct pathway*** Responder: recipient PBMC or CD4^+^ T cells Stimulator: Do and 3rdP PBMC Readout: IFNγ and IL-10 ELISpot Assay for regulation: Depletion and add-back of CD4^+^CD25^bright^ Tregs into MLR cultures Other assays: PBMC flow cytometry phenotype of CD4^+^CD25^hi^ FoxP3 Tregs	~33% patients developed donor-specific hyporesponsiveness 6–24 month post-tx with evidence of donor-specific Treg suppression	Function **Treg mediated donor-specific suppressive** function and hyporesponsiveness correlated with good graft function Phenotype Significantly **higher expression of FoxP3 in CD4^+^CD25^high^ T cells** detected in graft infiltrates of donor-specific hyporesponders than in non-hyporesponders
**Heart**; Dijke et al., 2009	ST (9) AR (12)^*^ ^*^Analysis before and during AR	***Direct pathway*** Responder: recipient CD4^+^CD25^neg/dim^ Stimulator: Do and 3rdP PBMC Readout: ^3^H proliferation Day 7 Assay for regulation: Depletion and add-back of CD4^+^CD25^bright^ Tregs into MLR cultures Other assays: PBMC flow cytometry phenotype of CD4^+^CD25^hi^ FoxP3 and CD127 Tregs	Pre-tx Treg suppressive function in ST group was significantly higher than detected in AR group *p* = 0.04 Treg mediated regulation of anti-donor responses was more significant in ST compared to AR in Treg depletion experiments *p* = 0.002 Add back of Tregs significantly suppressed donor-specific responses in ST group (vs. AR *p* = 0.001)	Function Preservation of Treg suppressive function correlated with improved graft survival Phenotype **No difference in % CD4^+^CD25^bright^ or FoxP3^+^ cells** in PB between ST and AR AR patients had higher % CD127^+^ within CD4^+^CD25^bright^ FoxP3^+^ T cells
**Kidney**; Sewgobind et al., 2008	ST (9) Analysis pre-tx to 0.5–2 years post-tx	***Direct pathway*** Responder: recipient CD4^+^CD25^neg/dim^ Stimulator: Do and 3rdP PBMC Readout: ^3^H proliferation Day 3 Assay for regulation: Add-back of isolated CD4^+^CD25^bright^ Tregs into MLR cultures Other assays: PBMC flow cytometry phenotype of CD4^+^CD25^bright^ Tregs	Donor-specific hyporesponsiveness by CD4^+^CD25^−/dim^ post-tx vs pre-tx *p* = 0.08 Detection of donor-specific Treg mediated regulation when added back as low ratios CD4^+^CD25^bright^ 1:20 CD4^+^CD25^−/dim^ *p* = 0.03	Function Development of donor-specific hyporesponsiveness and increase in **donor-specific Treg suppression** post-transplantation Phenotype No difference in % CD4^+^CD25^bright^ or FoxP3^+^ cells between ST and AR
**Kidney**; Game et al., 2003	ST (12) 2–20 years HC (12)	***Direct pathway*** Responder: recipient CD4^+^CD25^neg/dim^ Stimulator: Do and 3rdP PBM Readout: LDA ^3^H proliferation, IFNg ELISpot, IL-2 production Assay for regulation: Depletion of CD4^+^CD25^+^ Tregs from MLR cultures	ST: 8 of 11 patients showed donor-hyporesponsiveness by proliferation assay and 11 of 12 patients by IL-2 detection assay 1 patient showed evidence of non-donor specific Treg suppression	Function Donor-specific hyporesponsiveness associated iwth stable graft function No evidence of donor-specific Treg regulation Phenotype No differences in % CD4^+^CD25^+^ cells in PB between HC and ST
**Kidney**; Kreijveld et al., 2007	ST (14)^*^ 4–36 weeks ^*^5 patients used for functional assays	***Direct pathway*** Responder: recipient PBMC Stimulator: Do and 3rdP PBMC Readout: ^3^H proliferation Day 6, IFNg ELISpot, IL-2 production Assay for regulation: Add back of CD4^+^CD25^+^ Tregs into MLR	ST: 2 of 5 patients showed evidence of donor-specific Treg suppression	Function **Donor-specific Treg function** in 40% of stable patients
**Kidney**; Salama et al., 2003a	ST (15) 3 months–9 years AR (8) 1–11 years	***Indirect pathway*** Responder: recipient PBMC Stimulator: Do and 3rdP mismatched HLA-DR peptides Readout: IFNγ ELISpot Assay for regulation: Depletion of CD4^+^CD25^+^ Tregs from cultures	ST: 6 of 15 patients are hyporesponsive to indirect pathway donor stimulation 8 of 17 patient assays showed evidence of Treg mediated regulation of donor-specific responses AR: 1 of 8 patients was hyporesponsive to indirect pathway donor stimulation, 0 of 8 showed Treg mediated hyporesponsiveness	Function Evidence of **Treg mediated suppression of donor-specific indirect responses** in ~47% of stable patients and 0% of patients with graft rejection
**Kidney**; Spadafora-Ferreira et al., 2007	ST (3) AR (4) CR (4)	***Indirect pathway*** Responder: recipient PBMC Stimulator: Do HLA-DR peptides Readout: ^3^H proliferation ***Direct pathway*** Responder: recipient PBMC Stimulator: Do PBMC/spleen cells ± IL-4/IL-10 Readout: ^3^H proliferation Assay for regulation: Addition of generated Treg lines from patients into MLR	T cell lines with indirect alloreactivity established from 3 patients by stimulation of PBMCs with donor cells (5 cell lines generated) or Donor matched HLA-DR peptides (4 cell lines) in the presence of IL-4 orIL-10 T cell lines generated by indirect allostimulation could suppress both indirect and direct-alloresponses and contained FoxP3^+^ subsets	Function T cell lines generated from patients show ability to suppress indirect and direct-alloresponses, not assayed for donor-specificity

Summary of clinical studies of solid organ transplantation which have examined the presence of Tregs and contribution of Tregs toward mediating donor-specific hyporesponsiveness to donor alloantigens presented via the direct or indirect pathways of alloantigen presentation.

Time post-tx^a^, time post-transplant at which patients were studied; ST, stable graft function and maintained on standard Immunosuppression; HC, healthy control; AR, acute rejection; CR, chronic rejection; MMF, mycophenolate mofoetil; rATG, recombinant anti-thymocyte globulin; SRL, sirolimus; Do, donor cells; 3rdP, completely HLA mismatched cells to donor/recipient; 4thP, cells matched to donor/recipient HLA mismatched antigens; ^3^H proliferation, Thymidine incorporation measurement of proliferation.

Indeed, studies of human Tregs in patients with operational tolerance are more revealing and in general bode well for cell therapy approaches aiming to increase *in vivo* Treg numbers (Table 2, Column A). Several studies have detected increases in percentages of CD4^+^CD25^+^ Tregs as a proportion of total CD4^+^ T cells and also absolute numbers of Tregs in peripheral blood circulation in tolerant liver transplant recipients compared to healthy controls, patients with stable graft function whom are maintained on immunosuppressive drugs, and patients with active immune-mediated graft rejection (Li et al., 2004, 2008; Martinez-Llordella et al., 2007; Pons et al., 2008). Li et al. have further confirmed that higher percentages of FoxP3^+^ cells are also detected in biopsy material from some tolerant liver transplant patients (Li et al., 2008). More recently, expansion of *in vivo* numbers of circulating Tregs has been strongly linked to immunosuppression withdrawal protocols and establishment of tolerance in cohorts of liver transplant recipients (Nafady-Hego et al., 2010). The association of Tregs with operational tolerance in other organs such as kidney is not as consistent, with the majority of studies observing that tolerant patients do not have higher percentages or absolute number of Tregs in circulation compared to other patient groups described (Louis et al., 2006; Braudeau et al., 2007; Newell et al., 2010; Sagoo et al., 2010). In two recent studies examining the largest cohorts of renal transplant patients with established long-term operational tolerance to date, neither study detected expansion of CD4^+^CD25^high^ Tregs in peripheral blood (Newell et al., 2010; Sagoo et al., 2010). While all clinical studies of renal transplant recipients described in Table 2 identified no differences in percentages or numbers of circulatory Tregs between tolerant individuals and healthy control subjects, two studies did detect a significant reduction of Tregs in patients with chronic graft rejection (Louis et al., 2006; Braudeau et al., 2007). This observation suggests that tolerance may not be directly related to a numerical advantage in peripheral Tregs, more rather that tolerant individuals may maintain Tregs numbers similar to that of healthy individuals, whereas reduced Treg numbers may be associated with poor graft outcome. This difference may of course be a consequence of patients whom go on to develop chronic rejection having lower pre-transplant frequencies of Tregs, which is an important question that can be examined by the prospective and longitudinal immune monitoring of transplant recipients. Reviewing the clinical studies of operational tolerance summarized in Table 2 highlights an emerging dichotomy between liver and kidney transplantation and the differing role of circulatory Tregs within each organ transplant setting. This deserves further investigation and warrants deeper phenotypic and functional analysis, particularly in view of some divergence in genetic profiles of immunological tolerance that have recently been identified between these two organs (Martinez-Llordella et al., 2008; Perucha et al., 2011; Sawitzki et al., 2011).

**Table 2:** **Observational and functional study of Tregs in clinical operational transplantation tolerance**.

		**Column A**	**Column B**	**Column C**	**Column D**
**Organ Transplant**; **Reference**	**Study Groups (*n* =) time post-tx^a^**	**Methods for Treg detection & findings**	**Antigen-specific response studied & analysis method**	**Alloreactivity & evidence for Treg-mediated suppression**	**Summary & additional findings**
**Kidney**; Louis et al., 2006	^*^DF-Tol (4) CR (10) ST (12) HC (9) ^*^Stable function, sCRT <160 μmol/L >2 years IS-free (2–17)	% CD4^+^CD25^hi^ Tregs in PB: DF-Tol vs. HC = ns DF-Tol vs. CR *p* < 0.05 DF-Tol vs. ST *p* < 0.05 Increased foxp3 mRNA in PB: DF-Tol vs. HC = ns DF-Tol vs. CR *p* < 0.05 DF-Tol vs. ST = ns	Not done	Not done	Maintenance of **circulating Treg numbers is associated with tolerance**
**Kidney**; Sagoo et al., 2010	Training set patients ^*^DF-Tol (11) Mono (11) CR (9) ST-CNI (30) ST-nCNI (10) HC (19) ^*^Stable function, sCRT <160 μmol/L >1 year (1–21) Test set patients ^**^DF-Tol (24) Mono (11) CR (20) ST (34) HC (31) ^**^Stable function, sCRT 25% of baseline >1yr	% CD4^+^CD25^hi^ Tregs in PB: Training set DF-Tol vs. all = ns Test set DF-Tol vs. all = ns Increased foxp3 mRNA in PB: Training set DF-Tol vs. HC *p* < 0.01 DF-Tol vs. rest = ns Test set DF-Tol vs. HC = ns DF-Tol vs. ST *p* < 0.001	***Direct pathway*** Responder: selected recipient CD4^+^CD25^−^ and CD8^+^ T cells Stimulator: Do and 3rdP APCs Readout: IFNγ ELISpot Assay for regulation: Depletion of CD4^+^CD25^+^ Tregs from cultures ***Indirect pathway*** Responder: recipient PBMC Stimulator: Do and 3rdP membrane preps Readout: IFNγ ELISpot Assay for regulation: Depletion of CD4^+^CD25^+^ Tregs from cultures	Direct pathway donor-specific hyporesponse: Training set DF-Tol vs. Mono *p* < 0.05 DF-Tol vs. ST-nCNI *p* < 0.05 DF-Tol vs. ST-CNI *p* < 0.01 DF-Tol vs. CR = ns Test set DF-Tol vs. All = ns No evidence of Treg mediated regulation to direct pathway hyporesonsive state No detection of indirect pathway donor-specific hyporesponsiveness in any patient group	**Direct pathway donor-specific hyporesponses** associated with tolerance, not mediated by Tregs Tolerance associated with **B cell** bias in expression and phenotype
**Kidney**; Newell et al., 2010	^*^DF-Tol (25) ST (33) HC (25) Min 1 years (1–32) ^*^serum CRT within 25% of baseline	% CD4^+^CD25^hi^ Tregs in PB: DF-Tol vs. all = ns Increased foxp3 mRNA in urine sediment: DF-Tol vs. HC *p* < 0.01 DF-Tol vs. rest = ns	Not done	Not done	Tolerance associated with **B cell expression and phenotype signature**
**Liver**; Yoshizawa et al., 2005	^*^DF-Tol (14) HC (14) ^*^Weaned off IS	Not done	***Direct pathway*** Responder: recipient CD4^+^ T cells Stimulator: Do and 3rdP APCs Readout: CFSE and Proliferation Assay for regulation: Depletion of CD4^+^CD25^+^ Tregs from cultures	All patients showed donor-specific hyporesponsiveness. Mild donor-specific suppression by Tregs was detected in 4 of 5 patients	**Direct pathway donor-specific hyporesponses** associated with tolerance, Tregs only partially contribute to suppression of donor-specific responses
**Kidney and Liver** VanBuskirk et al., 2000	^*^DF-Tol (2) ST (8) AR and CR (4) ^**^Liv DF-Tol (1) Liv ST (4) ^*^Stable kidney function IS-free for 5 and 27 years ^**^Stable liver function IS-free for 5 years	Not done	***Indirect pathway*** Responder: recipient PBMC Stimulator: Do PBMC/spleen cell sonicates, HLA single antigen luminex beads and EBV antigen Readout: Trans-vivo DTH Assay for regulation: *in vivo* linked suppression assay of EBV response ± Do antigen ± TGFβ or IL-10 neutralising antibodies	Tol-DF patients showed donor-specific hyporespoiveness to indirect pathway, and donor-specific regulation which was TGFβ or IL-10 dependent	**Indirect pathway donor-specific hyporesponse** associated with tolerance, **with IL-10 or TGFb dependent** donor-specific regulation
**Kidney**; Braudeau et al., 2007	^*^DF-Tol (7) ST (15) CR (22) HC (10) ^*^ Stable function sCRT <150 lmol/l and proteinuria < 1 g/24 h IS-free for > 1 year (2–17)	Absolute CD4^+^CD25^hi^FoxP3^+^ Tregs in PB: DF-Tol vs. HC = ns DF-Tol vs. CR *p* < 0.05 DF-Tol vs. ST = ns CD4^+^CD25^hi^ Treg suppression of polyclonal stimulus: DF-Tol vs. All = ns	Not done	Not done	Maintenance of **circulating Treg numbers is associated with tolerance** and normal suppressive functions, which are IL-10/TGFβ independent
**Liver**; (Li et al., 2008)	^*^DF-Tol (28) ST (29) CR (7) HC (12) ^*^Stable function (47 ± 29 mths)	Intragraft FoxP3^+^ cells: DF-Tol vs. ST *p* = 0.0292 DF-Tol vs. CR *p* = 0.0128 DF-Tol vs. HC *p* = 0.0131 Intragraft biopsy mRNA foxp3 expression: DF-Tol vs. ST *p* = 0.07 DF-Tol vs. CR = ns DF-Tol vs. HC *p* < 0.0001	Not done	Not done	**Elevated intragraft Tregs** in some tolerant patients **Elevated intragraft *foxp3* mRNA** expression doesn't distinguish tolerance
**Liver**; Martinez-Llordella et al., 2007	^*^DF-Tol (16) ^**^ST (16) HC (10) ^*^>1 year IS free (1–6 years) ^**^Patients were weaned but returned to IS on signs of rejection	Higher % CD25^+^ in CD4^+^CD62L^hi^ in PB: DF-Tol vs. ST *p* < 0.0166 DF-Tol vs. HC = ns Higher % FoxP3^+^ in CD4^+^CD62L^hi^ in PB: Tol vs. ST *p* = 0.0151 DF-Tol vs. HC = ns	Not done	Not done	**Elevated circulating Tregs associated with tolerance**
**Liver**; Li et al., 2004	^*^DF-Tol (12) ST (19) HC (24) ^*^Normal CRP, AST, ALT, T-Bil, >1 yr	Higher % CD4^+^CD25^hi^ in PB: DF-Tol vs. ST *p* < 0.01 DF-Tol vs. HC *p* < 0.05 Higher absolute number of CD4^+^CD25^hi^ in PB: DF-Tol vs. ST *p* < 0.01 DF-Tol vs. HC = ns	Not done	Not done	**Elevated circulating Tregs and B cells** associated with tolerance
**Liver**; Nafady-Hego et al., 2010	^*^DF-Tol (24) ^**^ST-failed Tol (18) ^***^W (10) HC (17) ^*^Paediatric liver Tx recipients (7.3 ± 2.8 years) ^**^Failed weaning, returned to IS ^***^Active weaning	PB Flow cytometry of Tregs, conventional CD4^+^CD25^hi^CD45RA^−^ and naïve CD4^+^CD25^hi^CD45RA^+^: Conventional Tregs DF-Tol vs. HC *p* = 0.018 DF-Tol vs. ST *p* = 0.1 DF-Tol vs. W = ns Naive Tregs DF-Tol vs. ST *p* < 0.001 DF-Tol vs. rest = ns Higher % Foxp3^+^ cells in naive Tregs: DF-Tol vs. ST *p* = 0.048 DF-Tol vs. HC *p* = 0.032 DF-Tol vs. W = ns	***Direct pathway*** Responder: recipient CD4^+^ T cells depleted of Tregs Stimulator: Do and 3rdP PBMC Readout: ^3^H Proliferation Assay for regulation: Depletion of naive and conventional Tregs from cultures	Significant donor-specific hyporeonsivessness in DF-Tol group: DF-Tol vs. HC *p* = 0.037 DF-Tol vs. ST *p* = 0.045 DF-Tol vs. W = ns Only DF-Tol group demonstrated Treg-mediated donor-specific suppression by Conventional Tregs: DF-Tol vs. HC *p* = 0.007 DF-Tol vs. ST *p* = 0.017 DF-Tol vs. W = ns Naive Tregs: DF-Tol vs. HC *p* = 0.05 DF-Tol vs. ST *p* = 0.006	**Direct pathway donor-specific hyporesponse** associated with tolerance **Elevated naïve Tregs generated in periphery** are associated with tolerance and weaning **Donor-specific suppression by conventional and naive Tregs** associated with tolerance
**Liver**; Pons et al., 2008	Immune monitoring of 12 patients during IS withdrawal ^*^DF-Tol (5) AR (7) ST (19) HC (9) ^*^Maintained on CSA, for >2 years before complete IS withdrawal for >1 yr	PB Flow cytometry CD4^+^CD25^hi^: Progressive increase in % and absolute numbers of CD4^+^CD25^+^ and CD25^hi^ and *foxp3* mRNA expression in DF-Tol but not AR	Not done	Not done	**Elevated circulating Tregs and FoxP3** expression is associated with tolerance and weaning
**Liver**; Takatsuki et al., 2001	DF-Tol (13) (3–69 months)	Not done	***Direct pathway*** Responder: recipient PBMC pre and post-Tx Stimulator: Do and 3rdP APCs Readout: ^3^H Proliferation Assay for regulation: Not done	Donor-specific hyporesponsiveness detected in all except one patient, even though they maintained good function	**Direct pathway donor-specific hyporesponses in tolerance** Reduced IFNγ production detected in MLR cultures on Do stimulation (not detected for IL-10)
**Kidney**; Haynes et al., 2012	^*^ISO-DF-Tol (2) ^*^DF-Tol (11) Mono (7) ST (18) CR (7) ^*^sCRT within 25% of baseline >1 year	Not done	***Indirect pathway*** Responder: recipient PBMC Stimulator: Do PBMC/spleen cell sonicates, HLA single antigen luminex beads and Tetanus or Diptheria toxin Readout: Trans-vivo DTH Assay for regulation: *in vivo* linked suppression assay of TTOX/DT antigen ± Do antigen, ±neutralising TGFβ/IL-10 antibodies	Spectrum of indirect alloresponses lowest>highest DF-Tol-vs. Iso-Tol = ns DF-Tol vs. Mono *p* < 0.05 DF-Tol vs. ST *p* < 0.01 DF-Tol vs. CR *p* < 0.001 DF-Tol have highest level of regulation of indirect alloresponses: mean % inhibition of recall response in presence of Do antigen DF-Tol = 49 ± 19 Mono = 38 ± 20 ST = 41 ± 26 CR = 3 ± 3.8	**Indirect pathway donor-specific hyporesonsiveness** associated with tolerance **TGFβ-dependent mechanism of regulation** **Elevated naïve B cell numbers** associated with DF/ISO-Tol patients, although regulation to indirect pathway was B cell independent

Summary of research examining the association and function of Tregs in clinical studies of operational tolerance in solid organ transplantation.

DF-Tol, Drug-free tolerant; ^*^Tolerance indicator/period (IS) immunosuppression-free (range years); ISO-DF-Tol, Isogenic twin donor/recipient with established tolerance; Time post-tx^a^, time post-transplant at which patients were studied; ST, stable graft function and maintained on standard Immunosuppression; HC, healthy control; AR, acute rejection; Mono, monotherapy, e.g., maintenance prednisolone; CR, chronic rejection; MMF, mycophenolate mofoetil; rATG, recombinant anti-thymocyte globulin; SRL, sirolimus; Do, donor cells; 3rdP, completely HLA mismatched cells to donor/recipient; 4thP, cells matched to donor/recipient HLA mismatched antigens; CRP, C-reactive protein; AST, aspartate transaminase; ALT, alanine transaminase; T-Bil, total-bilirubin.

As suggested earlier, analysis of Treg frequencies in peripheral blood may be entirely perfunctory, and may not be indicative of allograft infiltrating Tregs or active mechanisms of regulation taking place within the tissue. Interestingly, despite observing no differences in peripheral blood Treg numbers between patient study groups, by studying urine sediment, which is anticipated to be reflective of the cellular composition of the kidney allograft, Newel et al. were able to detect higher *foxp3* expression by operationally tolerant patients compared to healthy control subjects, highlighting the subtleties of immunological monitoring that need to be considered when interpreting observations in clinical transplantation (Newell et al., 2010). Although intragraft Treg composition is challenging to measure directly, an intermediate method of assessing the role of Tregs and immune regulation in tolerance is through the detection of donor-specific hyporesponsiveness by allograft recipients.

### Evidence for donor-specific Treg-mediated regulation of alloimmunity

Measuring recipient alloreactivity toward donor antigen presented by the direct or indirect pathways has been achieved using several *ex vivo* methods as summarized in Tables 1 (Column A) and 2 (Column B), and has evidenced that detectable active donor-reactive immunity is in general correlated with poor graft outcome and development of acute and chronic graft rejection, respectively (Table 1 Column B and Table 2 Column C; Vella et al., 1997; Ciubotariu et al., 1998; Poggio et al., 2004; Hernandez-Fuentes and Lechler, 2005). Furthermore, by comparing recipient T cell responder frequencies against donor stimulation to that of HLA mismatched third party (3rdParty) stimulation, it can be used as a method to detect donor-specific hyporesponsiveness and, therefore, determine whether established stable graft function or immunological tolerance is alloantigen-specific. Monitoring of donor-reactive immune responses has demonstrated that hyporesponsiveness to the direct pathway can develop shortly after solid organ transplantation and is in general associated with stable graft function (Hornick et al., 1998; De Haan et al., 2000). Donor-specific cytotoxic or T cell hyporesponsiveness has been detected in kidney (Ghobrial et al., 1994; Mason et al., 1996; Mestre et al., 1996; Hornick et al., 1998; Baker et al., 2001b), heart (Hu et al., 1994; Hornick et al., 2000; Van Hoffen et al., 2000) and lung (De Haan et al., 2000) transplantation, although this is not a universal finding (Eberspacher et al., 1994; Loonen et al., 1994; Steinmann et al., 1994; Oei et al., 2000). In addition, hyporesponsivessness to donor antigen presented by the indirect pathway has also been described (Salama et al., 2003b). As shown in Tables 1 and 2, the contribution of Tregs toward regulation of donor alloreactivity can be measured by the recovery of effector functions or proliferation when Tregs are removed or added into an assay in which donor alloantigen is used as a stimulator of either the direct or indirect T cell alloresponse. Again, comparison of suppressive activity toward a 3rdParty stimulator can further reveal whether Tregs have alloantigen-specific suppressive functions.

Our review of clinical studies which have examined the contribution of Tregs toward established donor-hyporesponsiveness shows a high degree of concordance in demonstrating donor-specific Treg mediated suppression of T cell alloreactivity toward direct (Velthuis et al., 2006, 2007; Bestard et al., 2007; Kreijveld et al., 2007; Akl et al., 2008; Sewgobind et al., 2008; Hendrikx et al., 2009) and indirect (Salama et al., 2003b; Spadafora-Ferreira et al., 2007) pathway anti-donor responses in patients with stable allograft function. Two of these studies were prospective and were, therefore, able to show that donor-specific Treg activity with direct pathway specificity developed relatively early on post transplantation from 6 months onwards (Bestard et al., 2007; Hendrikx et al., 2009). Within the studies examined the percentage of stable allograft recipients with detectable evidence of donor-specific Treg functions ranged from 20 to 83% of donor-hyporesponsive patients between study cohorts. Some studies however, were either unable to detect any donor-specific T cell hyporesponsiveness (Baan et al., 2007), or unable to uncover any donor-specific Treg activity despite patients showing donor-specific T cell hyporesponses to direct pathway donor stimulation (Game et al., 2003). These variations may be in part explained by the differing immunocompetence and immune status between individual patients and within patient cohorts based on their pre- and post-transplant immunosuppressive regimens, or simply technical peculiarities of the assays performed. Incongruence's may also be influenced by differences in time post-transplantation that patients were screened for donor-specific Treg activity, where in addition to Tregs, other more dominant donor-specific immunoregulatory mechanisms may be actively contributing toward the detected dampening of anti-donor responses.

In support of this latter hypothesis, a study examining regulation of direct pathway alloreactivity in tolerant liver transplant recipients (Yoshizawa et al., 2005), suggests that donor alloantigen specific Treg activity is one of multiple mechanisms that may contribute to the maintenance of liver graft survival. Yoshizawa et al. were able to detect an increase in donor-directed alloreactivity after depletion of Tregs from *in vitro* MLR assays, however tolerant recipient T cells still remained largely hyporesponsive to donor stimulation suggesting other mechanisms such as clonal deletion, induction of donor-specific cell anergy or involvement of other immunomodulatory cell subsets were established during tolerance. Further evidence for this is provided by work from our group as part of the Indices of Tolerance and Riset consortium, where we were able to correlate direct pathway donor-specific hyporesponsiveness with tolerance in renal transplant patients, but this could not be attributed to CD4^+^CD25^+^ Treg mediated donor-specific functions by *ex vivo* analysis (Sagoo et al., 2010). As increased numbers of Tregs were more strongly associated with operational tolerance in liver transplantation as described earlier, the findings by Nafady-Hego et al. may be readily anticipated (Nafady-Hego et al., 2010). In their *ex vivo* functional assays to study donor-specific Treg suppression, Nafady et al. found that only the patient cohort with established tolerance demonstrated donor-specific Treg mediated regulation of donor-directed T cell alloresponses, with patients undergoing active weaning showing a similar emerging although not significant effect.

Drawing any firm conclusions from these findings is a challenge as very few studies to date have directly assessed Treg activity in clinical transplantation tolerance. However, the data reviewed in Tables 1 (Columns B and C) and 2 (Columns C and D) clearly indicate that donor-specific hyporesonsivess to the direct and indirect pathways of alloantigen presentation are features of stable graft function and also operational tolerance. Furthermore they suggest that naturally occurring Tregs may play a prominent role in the establishment and possibly maintenance of donor-specific immunological tolerance in liver transplantation. This is an important aspect which can only be assessed by clinical studies which perform longitudinal immune monitoring of transplant recipients during immunosuppression weaning protocols as is more routinely performed in liver transplantation. In contrast, while in kidney transplantation Tregs with direct pathway donor-specificity appear to contribute toward suppression of donor-specific responses during stable graft function or the phase of tolerance induction, they do not appear to play a prominent role in toward the maintenance of established operational tolerance.

Although there is evidence of Treg-mediated regulation of indirect pathway directed anti-donor responses in stable renal transplant patients (Salama et al., 2003a), clinical studies of operational tolerance suggest that maintenance of the immunological donor-specific hyporesponsive state can also be attributed to other immuoregulatory processes than naturally occurring Tregs. Several years ago, VanBuskirk et al. were able to demonstrate that some patients displaying stable tolerance to either a kidney or liver allograft had evidence of donor-specific regulation of indirect pathway T cell responses. This was detected using a trans-vivo delayed-type hypersensitivity (DTH) linked suppression assay, measuring DTH swelling responses to tetanus toxin recall antigen stimulation when applied with or without donor antigen (VanBuskirk et al., 2000). This study identified regulation of donor-specific responses was dependent on either IL-10 or TGFβ, although no further analysis of the mechanistic basis or source of either cytokine was studied. Following on from this work, Haynes et al., have recently been able to correlate both reduced T cell responses to indirectly presented donor alloantigens and evidence of active regulation of indirect alloreactivity with improved kidney transplant outcome (Haynes et al., 2012). Again using the trans-vivo DTH assay, they were able to show that tolerant patients had reduced donor-specific T cells responses with reactivity toward indirectly presented donor antigens, which were actively regulated through a TGF-β dependent mechanism detected using an *in vivo* linked suppression assay. Although again, immune regulation was not directly attributed to antigen-specific naturally occurring FoxP3^+^ Tregs or any particular lymphocyte subset, this certainly implies a role for adaptive or induced Th3 Treg mediated regulation, by CD4 or possibly CD8 T cell subsets. In this study, the importance of alloimmune priming and immune regulation of the indirect pathway for tolerance induction was also very clearly illustrated by the complete absence of indirect pathway alloresponses detected in identical twins receiving isogenic kidney transplants. Haynes et al. also performed a parallel assessment of other kidney graft patients including those with stable graft function and those undergoing chronic rejection, and showed a progressive spectrum of immune responses to the indirect pathway and the degree of active regulation of this alloresponse, associated with graft function, where patients with biopsy proven chronic rejection had the highest level of indirect alloreactivity, with the lowest regulatory ability.

These studies highlight the significance of active processes of regulation of the indirect pathway of alloantigen presentation in tolerance, suggesting that deletion may not be the primary mechanism involved, however they did not examine and, therefore, exclude the role of naturally occurring CD4^+^CD25^high^FoxP3^+^ Tregs, either their direct suppressive activities or their ability to mediate linked suppression and thereby induce alternative networks of immunomodulatory cells. Indeed, the clinical studies of operational tolerance reviewed here are all restricted by their assessment of only limited immunoregulatory cellular phenotypes or mechanisms. Nonetheless, what can be inferred from these studies is that in both liver and kidney transplantation tolerance, naturally occurring Tregs with donor-specificity may be related to immune regulation of the alloresponses early post-transplantation, to induce transplantation tolerance. There then appears to be some divergence between allografted tissues which requires further investigation, however current data suggests that in operational kidney transplantation tolerance, immunoregulation mediated by Tregs does not remain a dominant mechanism. An emerging hypothesis is, therefore, that Tregs with indirect allospecificity may induce other immunosuppressive mechanisms through linked or bystander suppression to generate infectious tolerance which are involved in maintenance of the tolerant state. We can find evidence to support this hypothesis by examining the mechanisms and immunological factors identified following experimental and clinical transplantation tolerance induction protocols.

### Lessons on relevance of donor-specific Tregs from tolerance induction protocols

Linked suppression is a feature of immune regulation which can be elicited by Tregs with indirect allospecificity, whereby a Treg can encounter an APC presenting its specific MHC:peptide complex and can exert suppression upon other T cell responses, with specificity for other unrelated antigens also presented by the same APC, namely via the indirect pathway (Chen et al., 1996; Wise et al., 1998; Niimi et al., 2001). Mechanistically, linked suppression is achieved through many of the classical suppressive functions of Tregs described, such as modulation of APC functions to generate tolerogenic functions, Treg-T cell competition for space or ligands on the APC both of which can result in anergy, or local production of immunosuppressive cytokines or factors which modulate lymphocyte functions (Qin et al., 1993; Waldmann et al., 2006). In addition to induction of adaptive CD4^+^CD25^+^FoxP3^+^ Tregs, these mechanisms may result in the generation of other immunomodulatory cell subsets, such as the recently described CD8^+^CD28^−^ suppressor T cells (Suciu-Foca et al., 2003), Tr1 cells (Battaglia et al., 2006), or Th3 cells, the importance of which has been further underlined in transplantation tolerance by recent work from the Burlingham research group described earlier (VanBuskirk et al., 2000; Haynes et al., 2012). The presence of donor-specific Tregs may, therefore, act to dampen the inflammatory alloimmune response early on post-transplantation by suppressing alloimmune T effector responses for induction of transplantation tolerance. Once a tolerogenic environment is established *in vivo*, subsequent antigen presentation may occur predominantly via the indirect pathway within a tolerogenic environment, and depending on the degree of HLA mismatching, resulting in the expansion of naturally occurring Tregs, or the induction of adaptive or induced Treg populations, such as the Th3 to propagate infectious tolerance and maintain an operational tolerant state. This may explain the incongruence between clinical studies that have examined the presence and activities of Tregs with direct pathway allospecificity during the late transplantation period. Although it remains to be formally demonstrated in a clinical setting, several studies have implicated a direct link between Tregs and induction of other immunoregulatory processes, which is emphasized by the persistence of the indirect pathway and indirect allospecific Treg functions.

Review of experimental tolerance induction strategies have shown that in addition to clonal deletion, anergy and exhaustion, immune regulation mediated by Tregs form a common mechanistic basis in achieving indefinite graft survival, particularly using methods such as DST, tolerogenic DCs and also costimulatory blockade (Sykes, 2007). Many tolerance induction protocols have been shown to require active presentation of alloantigen through the indirect pathway (Yamada et al., 2001; Niederkorn and Mayhew, 2002; Rulifson et al., 2002). This corresponds to studies of allograft tolerance in mice, where Tregs have been described as being generated by indirect presentation, which can then exert their suppressive properties against donor alloantigen presented by the indirect pathway (Wise et al., 1998; Hara et al., 2001). Also as mentioned previously, the detection of effective immune regulation of donor alloreactivity to the indirect pathway in clinical studies is correlated with stable graft function and tolerance. As several studies have indicated that thymically derived naturally occurring CD4^+^CD25^+^ Tregs have a higher propensity for recognition of self-MHC and thus indirect allospecificity (Jordan et al., 2001; Apostolou et al., 2002; Romagnoli et al., 2002; Hsieh et al., 2004), it is, therefore, conceivable that presentation of alloantigen by the indirect pathway following transplantation results in the preferential activation and expansion of Tregs with indirect allospecificity. This may in part explain their improved efficacy in experimental models of indirect pathway antigen presentation compared to direct pathway antigen presentation (Sanchez-Fueyo et al., 2007), and support their role in the early induction phase of transplantation tolerance.

The requirement of the indirect pathway and the role of Tregs in tolerance induction are heavily implicated by tolerance induction protocols using DST or hematopoietic stem cell transplantation (HSCT) in experimental and clinical transplantation (Kishimoto et al., 2004). Generation of mixed chimerism as an approach to achieve tolerance to a solid organ transplant aims to generate both peripheral and central tolerance to the allograft, and has recently been extensively reviewed by Pasquet et al. (2011). Establishing mixed chimerism creates an *in vivo* situation where alloantigen presentation via the indirect pathway is significantly potentiated in activity and intensity and may, therefore, be more permissive to the promotion of indirect allospecific Tregs and tolerance induction. Le Guern et al. have provided experimental data which links the mixed chimerism approach for tolerance induction, with Treg induction and linked suppression. In a murine model of fully mismatched heart transplantation (C57BL/6→ CBA), recipient mice received an autologous BMT (I-A^k+^) which had been retrovirally transduced to express a single donor MHC Class II donor allele (I-A^b^), followed by a donor or 3rdParty heart allograft. This protocol resulted in the induction of donor-specific tolerance, in the complete absence of sustained immunosuppression, which was associated with immune deviation from a Th1 to Th2 predominate cytokine response and with no indications of chronic rejection associated vasculopathy (LeGuern, 2004; LeGuern et al., 2010). Furthermore, they were able to transfer protection against graft rejection to naïve recipients through CD4^+^CD25^+^FoxP3^+^ Tregs isolated from tolerant mice.

An impressive Phase II clinical study by Leventhal et al. (2012) has employed the mixed chimerism approach to show induction of stable operational tolerance in all their study patients that underwent HLA-mismatched combined HSCT and kidney transplantation. This study used a protocol which included transfer of pre-plasmacytoid tolerogenic DC graft facilitating cells in addition to HSC donor inoculum, which by *in vitro* and in experimental *in vivo* research had previously been shown to mediate induction of CD4^+^CD25^+^FoxP3^+^ donor-specific Tregs. In patients whom developed durable chimerism, an increase in the ratio of Tregs to T effectors was detected, along with lack of donor alloreactivity toward the recipient, which may explain the absence of GvHD within the cohort. An additional observation, which we discuss later, was the expansion of CD19^+^ B cells by a large proportion of the patients, occurring within the first year post-transplantation. Based on detectable responses to 3rd Party alloantigens *in vitro*, it is likely that established donor-specific immune modulation in these patients was mechanistically linked to the induction of donor-specific Tregs. In another study of combined BMT and kidney transplantation in HLA-mismatched individuals, Tregs were found to play a more dominant role early post-transplantation, being expanded in numbers in the periphery and demonstrating the development of donor-specific suppressive activity compared to pre-transplant function. However at 6–12 months post-transplantation, only some study patients displayed evidence of donor-specific suppression by Tregs, which was no longer detectable after 1 year, despite the maintenance of donor-specific hyporesponsiveness. This suggests other forms of immune regulation such as antigen-specific T effector cell depletion or anergy may have evolved to maintain allograft tolerance (Andreola et al., 2011). These studies suggest that maintenance of established tolerance may be manifested through CD4^+^CD25^+^FoxP3^+^ Treg dependent or independent activity, which may act in concert with or independent of several other induced immunomodulatory mechanisms.

The use of donor-specific Tregs for transplantation immunotherapy, therefore, provides an opportunity for the generation of infectious tolerance and as such, an improved likelihood of developing stable long-term donor-specific unresponsiveness (Cobbold and Waldmann, 1998; Waldmann et al., 2006). We next explore the concept of linked suppression in the context of the improved capacity of donor-specific Tregs to mediate maintenance of tolerance induction.

### Donor-specific Tregs and consequences of regulation through linked suppression

As described, a key advantage of immunotherapy using Tregs with indirect donor allospecificity is that they have the capacity to mediate linked suppression. This allows them to exert control over broader effector arms of the alloimmune response, which is particularly relevant for the control of alloantigen-specific B cells of the alloimmune response.

One of the main effector mechanisms of alloreactive T cells with indirect allospecificity is through the provision of T cell help to B cells, which results in the generation of the humoral alloantibody response to an allograft (Suciu-Foca et al., 1995; Colvin, 2007), and leads to alloantibody mediated chronic graft rejection. The dominant role of T cells with indirect pathway allospecificity in providing germinal center help to B cells for alloantibody induction and graft rejection has recently been firmly established. By adoptive transfer of CD4^+^ T cells with indirect allospecificity (BALB/c Class I H2-K^d^ molecule presented in the context of C57BL/6 class II I-A^b^) or T cells with direct allospecificity (CD4^+^ T cells with direct specificity for I-A^bm12^), Conlon et al., were able demonstrate that only T cells with indirect allospecificity had the capacity to provide B cell help and induce an alloantibody response to a heart allograft (BALB/c→C57BL/6), whereas T cells with direct allospecificity alone were incapable (BALB/cxBM12→C57BL/6; Taylor et al., 2007; Conlon et al., 2012). Interactions between cognate T cells and B cells, recognizing different allogeneic peptides presented by the same APC also promotes epitope spreading of the T cell and alloantibody responses, resulting in the recognition and alloimmune targeting of effector responses to more cryptic antigenic determinates over the lifetime of the transplant. This correlates with the main pathological markers of chronic rejection which are typically that of wound healing such as fibrosis or vasculitis, where proinflammatory cytokines generated by antibody-mediated complement or cellular dependent mechanisms of allograft damage, induce endothelial and epithelial hyperproliferation. Indeed in clinical transplantation, epitope spreading has been detected in patients with detectable indirect pathway T cell alloreactivity with evidence of chronic allograft dysfunction (Vella et al., 1997; Ciubotariu et al., 1998; Suciu-Foca et al., 1998; Hornick et al., 2000). Previous work from our laboratory has demonstrated the unique capacity of Tregs with indirect allospecificity to control alloantibody mediated vasculopathy in experimental heart and skin graft models (Tsang et al., 2009). Using both MHC-mismatched and semi-allogeneic transplantation models, Tregs generated with both direct- and indirect pathway allospecificities were found to be more effective at inducing indefinite survival of heart transplants than Treg cell lines generated for direct allospecificity alone. Whilst Tregs with allospecificity for the direct pathway were only marginally less effective at inducing indefinite graft survival compared to Tregs with both direct and indirect pathway allospecificities, Tregs with indirect allospecificity were essential to prevent chronic vasculopathy, determined by allograft histopathology. These findings correspond to clinical observations, where Tregs with indirect pathway donor-specific suppressive activity in stable renal transplant recipients have been shown to be able to regulate a shift in recipient alloreactivity to different donor MHC epitopes during the post-transplant period (Salama et al., 2003b).

These data clearly argue that development of Tregs with donor-specificity for the indirect pathway as a cell therapy product for transplant recipients is likely to be critical for the induction of long-term graft survival, enabling immunoregulatory mechanisms to adapt to the evolving immune response through the ability of Tregs to control epitope spreading through linked-suppression. Recipient B cell presentation of donor graft alloantigens is now known to make a critical contribution toward graft rejection (Noorchashm et al., 2006). As only Tregs with indirect specificity would have the capability of interacting directly with its cognate MHC: donor allopeptide complex as presented by a B cell, it would, therefore, have the potential to modulate the B cell alloresponse. Treg-mediated modulation of B cell activity has been evidenced in a previous study of MHC class I mismatched heart allograft rejection mediated by the indirect pathway, where graft rejection, induced specifically via CD4^+^ T cell dependent induction of alloantibody, was prevented using a tolerance induction protocol of anti-CD4 and DST, which was shown to generate Tregs with indirect allospecificity with the capacity to suppress alloantibody generation (Callaghan et al., 2007).

In addition to the indirect regulatory T cell effect on B cell activity, through inhibition of helper T cell activity, Tregs have also been shown to modulate B cell activity through a number of direct suppressive mechanisms. For example, antigen-specific murine Tregs raised against a common allergen, through *in vivo* administration of an immunodominant peptide, have been shown to mediate B cell killing on recognition of specific epitope:MHC complexes through Treg cytolytic activity of Fas-Fas-L interactions (Janssens et al., 2003). Lim et al. (2004) have shown that on antigen-mediated activation, Tregs can modulate their expression of germinal center (GC) B cell follicular zone homing chemokine receptors (increase CXCR5, decrease CCR7) and migrate to the T cell B cell boundary areas within human lymphoid tissue, where they can then directly prevent B cell class switching and Ig production (Lim et al., 2005). This latter study went further and by isolating Tregs and B cells from human lymphoid tissue, were able to demonstrate that Tregs were not only able to directly suppress B cell class switching, detected by monitoring Ig transcript analysis in *ex vivo* transwell assays, but were also able to suppress the helper T cell response, by preventing CXCL13 secretion. More recent studies in mouse have shown that naturally occurring Tregs can migrate and reside in the GC follicular zone where they regulate B cell humoral responses, and that inhibiting their migration to these zones (using B cells derived from CXCR5^−/−^ mice) results in aberrant B cell IgM, IgA, IgG1, and IgG2b antibody production (Wollenberg et al., 2011). More recently experimental evidence suggests that Tregs may also have the capacity to protect pre-sensitized individuals, through their ability to control plasma B cell activity (Jang et al., 2011). Tregs are, therefore, key in preventing B cell mediated graft destruction and limiting indirect alloimmunity, which correlates with an absence of donor-specific alloantibody in clinical transplantation tolerance and experimental models of transplantation tolerance.

Although the classic view of B cells in transplantation has focused on their pathogenic activities, an alternative emerging view is that of the complimentary roles of Regulatory B cells (Bregs) and Tregs. Bregs have recently been identified to play roles in regulating autoimmunity in experimental models of collagen-induced arthritis, experimental autoimmune encephalitis (EAE) and colitis, which is associated with B cell production of IL-10 (BR1 or B10 cells in human or mouse respectively) or TGFβ production (Mauri and Blair, 2010). Ashour and Niederkorn have demonstrated that Breg and Treg collaboration are associated with the process of immune modulation in anterior chamber associated immune deviation (ACAID). Their study showed that following antigen transfer into the anterior chamber of the eye, the APC function of B cells was essential in generating peripheral tolerance through the induction of CD4^+^ and CD8^+^ regulatory T cells (Ashour and Niederkorn, 2006). Further evidence is provided in a model of EAE, where depletion of B cells was related to a lack of recovery from the disease and delayed emergence of FoxP3^+^ cells within the CNS (Ray et al., 2012). Interestingly, in this model, B cell induction of Treg proliferation was found be dependent on B cell expression of glucocorticoid-induced TNF ligand (GITRL) rather than IL-10 expression. In fact, B cells are now becoming well described in their abilities to both positively and negatively regulate T effector responses, in addition to both induce and expand Tregs (Lund and Randall, 2010). Thus, an emerging controversial role of B cells in immunity is apparent, which is particularly surprising given the established contribution of allospecific B cells and alloantibody toward organ allograft rejection.

Currently very few studies have identified a role for Bregs in the context of transplantation tolerance (Le Texier et al., 2011; Wang et al., 2011). Le Texier et al. have recently revealed that B cells isolated from tolerant recipients in a rat model of heart transplantation can mediate infectious tolerance on adoptive cell transfer (Le Texier et al., 2011). In this model, B cells infiltrated and were localized to the tolerated organ, did not undergo class switching being maintained as IgM^+^ cells within the tissue and the periphery, and were found to express high levels of BANK-1 and the inhibitory FcgR2b receptor, indicating the generation of an inhibitory B cell phenotype. In clinical transplantation, the study by Haynes et al. mentioned earlier has been the first to examine the functional regulatory contribution of Bregs. In their study, they observed highest regulation of indirect pathway alloreactivity in operationally tolerant recipients which was predominantly TGFβ dependent. One of the methods they used to examine a Breg effect was by incorporating B cell depletion into their studies measuring the *in vivo* DTH response, which showed regulation of indirect alloreactivity was mediated through a B cell–independent mechanism. These two studies suggest that maintenance of established tolerance may be more dependent on B cells, rather than tolerance induction. One possibility may be that following immunosuppression withdrawal, Breg populations may emerge which are actively involved in mediating tolerance through other mechanisms such as IL-10 production (Iwata et al., 2011), which allows speculation that they then have the potential to promote other immunomodulatory mechanisms such as induction of Tr1 Tregs. These studies correspond with the B cell dominant gene expression profile and peripheral expansion of B cells detected within operationally tolerant patient cohorts (Newell et al., 2010; Pallier et al., 2010; Sagoo et al., 2010), some of which show alterations in specific memory or transitional B cell subsets. One of the main difficulties in assessing the role of Bregs in clinical transplantation tolerance is the current lack of a definitive Breg marker. In view of these findings, a resurgent interest in the regulatory and allopriming role of B cells in transplantation tolerance is occurring (Adams and Newell, 2012) which may identify a Breg subset phenotype. More research may also reveal whether Tregs can influence or alter the generation of a Breg or B cell repertoire composed of more tolerogenic anti-inflammatory subtypes post-transplantation. This and other questions raised throughout this review may only be answered by the sequential immunological monitoring of patients pre- and post-transplantation, operationally tolerant patients or patients undergoing tapered weaning protocols, as are currently being performed within the GAMBIT study (Genetic Analysis and Monitoring of Biomarkers of Immunological Tolerance) at King's College London UK.

## Summary and perspective

Achieving transplantation tolerance may be viewed as two interlinked phases, tolerance induction and maintenance of established tolerance. This review finds that while Tregs are associated with, and in some studies, integral to tolerance induction, Treg-mediated immune regulation may not be a consistent feature of long-term tolerance, although this may be an organ-specific phenomenon. To obtain a clearer perspective of the role of Tregs in the process of establishing tolerance, the evolving alloimmune response needs to be studied on a longitudinal basis in terms of several immunomodulatory population phenotypes, e.g., Treg, Tr1, Th3, Bregs, and functions, using an array of complementary methods, with parallel monitoring of clinical allograft function. This sort of comprehensive immune monitoring will require a solidly collaborative approach, but may reveal the true potential of Tregs for induction of transplant tolerance.

In developing an optimized Treg therapy for clinical induction of transplantation tolerance, Tregs with indirect donor-alloantigen specificity are likely to be most effective at delivering long-term stable graft survival, which is attributed to their ability to suppress multiple immune cell types, and their potential to interact with and promote other immunoregulatory processes, through linked suppression and infectious tolerance. New findings highlight an emerging role of the semi-direct pathway in alloantigen presentation, which combined with the prominent role of the indirect pathway in driving rejection and tolerance, makes a stronger case for the use of indirect allospecific Treg therapy. Indeed, although Tregs with direct allospecificity may be able to deliver localized immune regulation to the allograft, they would be limited in their ability to control the effector and allopriming arms of the indirect alloresponse, which occur at different anatomical sites, however this would again be dependent upon the degree of HLA matching between the donor and recipient. Furthermore, although direct allorecognition can lead to a vigorous inflammatory response resulting in direct cell mediated damage and hyperacute rejection of allografted tissues, it can be effectively controlled with immunosuppressive drugs to avoid acute rejection, as evidenced by the high success rate of graft acceptance early post-transplantation.

Clinical translation of Treg cell therapy faces several major challenges. First and foremost is the challenge of developing clinically transferrable protocols for the selection and expansion of human donor alloantigen-specific Tregs, particularly Tregs with indirect allospecificity (Jiang et al., 2006; Peters et al., 2008). Very little progress has been made in this respect, which is primarily due to the complexity of studying indirect allorecognition with the tools and methods currently available, which is further complicated by the breadth of mismatched allogeneic HLA peptides that alloreactive T cells may respond to (Waanders et al., 2008). The advantage is that by generating Tregs with specificity for a single immunodominant allopeptide, the Tregs will be able to mediate regulation against all allogeneic peptides through linked suppression as evidenced by the work described above (LeGuern et al., 2010). Indeed, work is currently underway in our laboratory to generate human Tregs with indirect allospecificity, to demonstrate this potential in an *in vivo* humanized mouse xenograft model.

A potentially serious caveat to using donor alloantigen-specific Tregs *in vivo* is the potential of transferring contaminating alloreactive T effectors, or indeed alloantigen-specific Tregs with the capacity to convert to proinflammatory Th17 effector cells, particularly in light of their emerging role in contributing toward graft rejection (Burlingham et al., 2007; Chadha et al., 2011). Efforts to limit this possibility are focusing on identifying key triggers and Treg subset markers which describe Tregs with Th17-conversion potential. However, do we really need to limit transfer of effector populations of Th17 differentiating cells or will the transfusion of Tregs into a regulatory or suppressive environment induced by immunosuppression for example, result in immunodominance by Tregs? Treg cell products currently being used in clinical HSCT can often be composed of only 50% FoxP3^+^CD4^+^ T cells, to deliver a Treg and graft versus leukemia (GvL) effect *in vivo*, with no adverse or aggressive GvHD effects reported as a direct consequence (Edinger and Hoffmann, 2011). Further *in vivo* studies are required to understand the real risk of donor-specific Treg infusion in cell therapy.

Another potential limitation of Treg therapy is assessing its capacity to control alloreactive memory. Memory T cell responses naturally provide rapid and potent T cell immunity and are a barrier to most transplantation tolerance induction strategies (Lakkis and Sayegh, 2003). Although Tregs have been demonstrated to work extremely effectively at inducing transplantation tolerance in murine models, very few have examined the capacity of Tregs to primed T cell responses (Marshall et al., 1996), to inhibit allograft rejection. Studying the efficacy of Tregs in experimental rodent systems and models cannot be accurately assessed due to the absence of a memory T cell pool, the importance of which has recently been demonstrated in a study correlating the presence or frequency of pre-existing T cell memory cells in non-human primates, with graft rejection (Nadazdin et al., 2011). The ability of Tregs to regulate memory T cell responses has been shown to be limited compared to naïve T cells when applied at the same ratio of Tregs to effectors cells (Yang et al., 2007; Afzali et al., 2011), which has important implications for the timing at which Treg therapy may need to be applied as pre-existing alloreactive memory T cells may otherwise be stimulated by the transplanted organ to provoke an aggressive alloimmune response (Brook et al., 2006). Treg therapy may need to be applied in concert or in succession to immunosuppression to efficiently overcome donor-specific memory T-cell responses, as recently demonstrated by Yamada et al. using a presensitised non-human primate model of combined renal allograft and mixed chimerism, to induce transplantation tolerance (Yamada et al., 2012). The humanized mouse also represents a useful tool which can be engineered as a clinically relevant model of human graft rejection to study human Treg function (Shultz et al., 2007; Nadig et al., 2010; Sagoo et al., 2011). By reconstituting mice with human PBMCs, human immune subsets, replete with memory compartments can be established to permit an assessment of the capacity of human Tregs to regulate memory immune subsets or processes in order to achieve transplantation tolerance. It may also allow a more comprehensive evaluation of the immunoregulatory effects of Tregs on a more complete spectrum of functional human immunity, such as priming of indirect alloresponses and B cell mediated alloimmunity.

In summary, review of experimental and clinical data on transplantation tolerance support the use of donor-specific Treg therapy for establishing immunological tolerance in the clinc, in particular, Tregs with indirect allospecificity. Although current studies lack a clear demonstration of the comparative efficacy of Tregs with either direct or indirect allospecificity, there is strong evidence for an integral role of Tregs in establishing tolerance, although their contribution toward maintaining the stable tolerant state is unclear, and requires further investigation. Treg cell therapy may, therefore, be envisaged as being administered early during the post-transplantation period to accelerate the generation of other associated immunoregulatory processes that act toward maintaining stable immunological graft acceptance.

### Conflict of interest statement

The authors declare that the research was conducted in the absence of any commercial or financial relationships that could be construed as a potential conflict of interest.
